# Interpretable machine learning-based decision support for prediction of antibiotic resistance for complicated urinary tract infections

**DOI:** 10.1038/s44259-023-00015-2

**Published:** 2023-11-02

**Authors:** Jenny Yang, David W. Eyre, Lei Lu, David A. Clifton

**Affiliations:** 1 Institute of Biomedical Engineering, Department Engineering Science, University of Oxford, Oxford, UK; 2 Big Data Institute, Nuffield Department of Population Health, University of Oxford, Oxford, UK; 3 Oxford-Suzhou Centre for Advanced Research (OSCAR), Suzhou, China

**Keywords:** Therapeutics, Translational research

## Abstract

Urinary tract infections are one of the most common bacterial infections worldwide; however, increasing antimicrobial resistance in bacterial pathogens is making it challenging for clinicians to correctly prescribe patients appropriate antibiotics. In this study, we present four interpretable machine learning-based decision support algorithms for predicting antimicrobial resistance. Using electronic health record data from a large cohort of patients diagnosed with potentially complicated UTIs, we demonstrate high predictability of antibiotic resistance across four antibiotics – nitrofurantoin, co-trimoxazole, ciprofloxacin, and levofloxacin. We additionally demonstrate the generalizability of our methods on a separate cohort of patients with uncomplicated UTIs, demonstrating that machine learning-driven approaches can help alleviate the potential of administering non-susceptible treatments, facilitate rapid effective clinical interventions, and enable personalized treatment suggestions. Additionally, these techniques present the benefit of providing model interpretability, explaining the basis for generated predictions.

## Introduction

Recent years have seen rapid increases in the prevalence of antimicrobial resistance in bacterial pathogens, which is threatening the efficacy of many antibiotic therapies, and ultimately leading to treatment failure^1–3^. Although new drugs are urgently needed, new antibiotic development is restricted by costs, limited government support, and regulatory requirements^1,2^. For instance, as of 2019, major pharmaceutical corporations, commonly known as “big pharma,” were progressively divesting themselves of antibiotic research and development (R&D) assets^4^. This shift restricts the opportunities available to smaller companies and their investors, leading to heightened financial constraints and a lack of infrastructure for antibiotic R&D.

Furthermore, antibiotic resistance leads to increased reliance on broad-spectrum therapies, which select for further resistance, exacerbating the issue at hand^3,5^. To avoid these risks, it is critical for clinicians to accurately align available antibiotic therapies with the precise susceptibilities of bacterial pathogens. Ideally, this alignment should occur when initiating empirical treatment, even before culture results are obtained (which might take several days to be available). In this study, we present interpretable machine learning (ML)-based methods for predicting antimicrobial resistance (AMR), which decreases the risk of non-susceptible and therefore, ineffective treatment, and facilitates rapid effective clinical intervention. We demonstrate the utility of these systems for urinary tract infections (UTIs), where the problem of antibiotic resistance is of particular importance.

UTIs are one of the most common bacterial infections worldwide, affecting more than 150 million people each year^3,6^. The pathogens that cause UTIs, including *Escherichia coli*, *Klebsiella pneumoniae*, *Proteus mirabilis*, *Enterococcus faecalis* and *Staphylococcus saprophyticus*^3,6,7^ can be carried asymptomatically and thus, are frequently exposed to antibiotics, including those intended for other infections^2^. This exposure, combined with high recurrence rates, often results in multidrug-resistant strains, with resistance rates of over 20% for commonly used drugs^3^. As treatment-outcome is associated with the infecting pathogen’s susceptibilities, clinicians are faced with the challenging task of correctly prescribing patients with the most appropriate antibiotics. However, to offer rapid intervention, treatment is commonly administered empirically, lacking insight into the specific antibiotics that the infecting pathogen may be susceptible to^2,3^. This scenario adds to the potential of choosing an inadequate treatment regimen.

Recent studies have shown that ML-based algorithms, using electronic health record (EHR) data, including demographic information, prior antibiotic exposures, prior microbiology antibiotic susceptibility data, basic laboratory values, and comorbidities, can be used to predict antibiotic resistance in UTI infections. Analyzing six different antibiotics, Yelin et al.^3^ demonstrated that logistic regression and gradient-boosting decision trees could effectively improve the predictability of resistance (AUROC range 0.70–0.83), using demographics, microbiology sample history and antibiotic purchase history. Subsequently, they also found that the algorithm-suggested drug recommendations reduced the rate of mismatched treatments, both when using an unconstrained method (where the antibiotic with the lowest resistance probability was chosen) and a constrained method (where antibiotics were selected at the same frequency used by clinicians). Although past purchase history was shown to have high predictive power, past antibiotic purchases and treatment can be associated with different clinical conditions including comorbities and hospitalizations, which were not considered in the study. Similarly, Kanjilal et al.^5^ used EHR data to predict the probability of antibiotic resistance for uncomplicated UTIs. They performed retrospective analyses on a subset of patients with uncomplicated UTI, consisting of 15,806 specimens. This uncomplicated cohort was defined as specimens where the infection site was specified as urinary, and the following patient criteria were met: female between the ages of 18 to 55, no diagnosis indicating pregnancy in the past 90 days, no selected procedure (placement of a central venous catheter, mechanical ventilation, parenteral nutrition, hemodialysis, and any surgical procedure) in the past 90 days, no indication of pyelonephritis, and exactly one antibiotic of nitrofurantoin, co-trimoxazole, levofloxacin, or ciprofloxacin prescribed). The trained models achieved AUROCs between 0.56–0.64 across four different antibiotics. Despite relatively modest predictive performance, this still out-performed clinicians. In addition to predicting resistance, they also aimed to reduce the recommendation of broad-spectrum second-line therapies (e.g., fluoroquinolone antibiotics such as ciprofloxacin and levofloxacin, which have also been associated with serious adverse events in some patients). Using logistic regression and post-processing analysis, they found that their pipeline both reduced inappropriate antibiotic recommendations and achieved a 67% reduction in the recommendation of second-line agents, relative to clinicians. Although these studies found that logistic regression and gradient-boosting trees achieved the best results, neither investigated the effectiveness of neural network-based architectures.

Deep neural networks have notably been used for tasks involving image- and text-based data. However, it remains underexplored for tabular data, as ensemble-based decision trees (DTs) have typically achieved state-of-the-art success for such applications. One reason for this is that deep neural networks are overparametrized; and thus, the lack of inductive bias results in them failing to converge to optimal solutions on tabular decision manifolds^8^. Furthermore, a DT is highly interpretable, whereas a deep neural network is less straightforward to interpret, even commonly being referred to as a “black box”^9^. This makes it difficult to implement neural networks for many real-world tasks, as model-interpretability is particularly important, especially for applications concerning clinical decision-making. However, there are many benefits to using neural networks, including improved performance on large datasets, and the ability to use transfer learning and self-/semi-supervised learning^8,10^. Moreover, with the advancements and increasing popularity of attention-based models (a type of sequence-to-sequence model), researchers have developed deep architectures capable of reasoning from features at each decision step, enabling model interpretability. One such model is the TabNet architecture^8^, which is uniquely tailored for interpretable learning from tabular data. During training, the model uses “sequential attention” to dynamically select relevant features at each step of the prediction, focusing on the most informative aspects of the input data for each specific task. This feature selection mechanism helps to reduce noise and unnecessary information, and has been shown to improve model performance and interpretability^8^.

With a focus on predicting antibiotic susceptibility, we aimed to expand on previous studies by (1) evaluating the utility of using ML-based prediction of antibiotic resistance for patients with potentially complicated UTIs (namely, UTIs which are more severe in nature, and/or occur in patients with anatomically abnormal urinary tracts or significant medical or surgical comorbidities^11^) and (2) demonstrating, comparing, and discussing the advantages of three types of interpretable machine learning architectures, including a neural network-based model (specifically, a TabNet architecture).

We chose to focus on potentially complicated UTIs, as these infections typically carry a higher risk of treatment failure due to prior antibiotic therapy, and are associated with more adverse outcomes with ineffective treatment. These infections may also require longer courses of treatment, different antibiotics, and varying degrees of intervention^12,13^, emphasizing the necessity for novel intervention methods. We specifically opted for interpretable machine learning algorithms, based on the unique importance of interpreting and elucidating model predictions in clinical settings. Such interpretability supports clinical utility and the integration of machine learning models into regular care practices by healthcare professionals.

Given our focus on a diverse and heterogeneous patient cohort with potentially complicated UTIs, our primary objective revolves around discerning antibiotic resistance to support clinicians in their decision-making process. The aim is to swiftly predict antibiotic resistance, rather than determining the necessity or type of antibiotic therapy. Hence, it remains imperative for clinicians (or another dedicated pipeline) to evaluate and ascertain the suitability of antibiotic therapy for each patient independently. While antibiotic resistance for complicated UTIs was the motivating problem, the techniques introduced can be applied to many other applications.

## Results

### Cohort summary

Patients in the training set cohort had a median age of 64 years (IQR 44–76), with 72.9% of patients self-identifying as white; the validation cohort also had a median age of 64 (44–76), with 73.6% self-identifying as white; and the test cohort had a median age of 64 (45–76), with 72.7% self-identifying as white. This differs from the uncomplicated UTI patient cohort presented in Kanjilal et al.^5^, who by definition were all female, and where the median age was 32 years (24–43), and 64.2% of patients self-identified as white (recall that the uncomplicated cohort specified an age range between 18–55). It should be noted that demographic information on the sex of patients in the complicated UTI cohort was not available. Patients in the complicated UTI test set cohort presented more frequently in the emergency room (27.8% compared to 19.6% for the test set and training set cohort, respectively). The prevalence of resistance to fluoroquinolones in the training and test set cohorts (with patient presentations between 2007–2013) was similar to national estimates reported in a cross-sectional survey in the United States in 2012^14^, which found that resistance was high among adults (11.8%) and elderly outpatients (29.1%) (compared to 21.6–24.7% for training, validation, and test cohorts used in our study). For first-line therapies, the prevalence of resistance to SXT was similar to those reported in the study (22.3% and 26.8% for adults and older adults, respectively; compared to 22.3–23.6% in our cohorts); however, the prevalence of resistance to NIT in our cohorts was higher (0.9% and 2.6% for adults and older adults, respectively; compared to 22.3–22.5% in our cohorts). The majority of patients in our training, validation, and test cohorts had no prior drug resistant infections, recorded within the previous 90 days of the specimen sample (6.8–6.9%, 6.5–6.7%, 7.8–9.0%, and 9.1–9.7% for prior NIT, SXT, CIP, and LVX resistances, respectively, across training, validation, and test cohorts). A full summary of baseline characteristics for the training, validation, and test sets are presented in Supplementary Table 2.

### Model performance

We individually trained LR, XGBoost, TabNet, and TabNet^self^ models for each antibiotic; thus, training and test data slightly differed depending on whether a patient had susceptibility results for the antibiotic being tested. A summary of all training, validation, and test cohorts can be found in Table 1.

**Table 1:** Summary of the total number of specimens and non-susceptible cases in training, validation, and test set cohorts, for each antibiotic susceptibility prediction task.

		NIT	SXT	CIP	LVX
Training	n, specimens	58,972	53,865	57,631	61,586
	n, non-susceptible (%)	13,925 (23.6%)	13,851 (25.7%)	13,495 (23.4%)	15,123 (24.6%)
Validation	n, specimens	6553	5986	6404	6843
	n, non-susceptible (%)	1514 (23.1%)	1561 (26.1%)	1492 (23.3%)	1711 (25.0%)
Test	n, specimens	30,528	27,997	30,920	31,690
	n, non-susceptible (%)	7138 (23.4%)	7536 (26.9%)	7637 (24.7%)	7907 (25.0%)

After training models on cohorts of patients diagnosed with complicated UTI between 2007 and 2013, we temporally validated our models on patients diagnosed with complicated UTI between 2014 and 2016. Separate sets of models were trained to predict resistance for each of four antibiotics – NIT, SXT, CIP, and LVX (Table 2). Overall, higher predictive performance was achieved by models developed for the second line antibiotics – CIP and LVX (mean AUROCs across all models of 0.800 [95% CIs ranged from 0.784–0.916] and 0.804 [0.786–0.810], respectively), than the first line antibiotics – NIT and SXT (0.674 [0.656–0.681] and 0.686 [0.660–0.707], respectively). For all antibiotics, XGBoost models achieved the best performances with respect to both AUROC and AUPRC. LR and TabNet (without pre-training) models achieved the lowest AUROC and AUPRC scores, with non-overlapping CIs (except for the AUPRC CIs for the NIT model) when compared to XGBoost comparators, across all antibiotics, suggesting meaningful improvements were obtained through using the XGBoost architecture (*p* < 0.001 across all antibiotics; p-value calculated by evaluating how many times XGBoost performs better than other models across 1000 pairs of iterations). However, when the TabNet models were pre-trained using a self-supervised method (TabNet^self^), AUROC and AUPRC scores improved across all antibiotics. Although overall predictive performance between TabNet^self^ and XGBoost models were similar, TabNet^self^ did not outperform the XGBoost comparators (*p* < 0.001 for all antibiotics).

**Table 2:** Performance metrics, alongside 95% confidence intervals, for antibiotic resistance prediction for patients with complicated UTI (bolded values denote best [^a^] and second-best [^b^] scores for AUROC and AUPRC).

Antibiotic	Model	AUROC	AUPRC	Sensitivity	Specificity	PPV	F1
NIT	LR	0.662(0.656–0.668)	0.381 (0.371–0.390)	0.623 (0.614–0.633)	0.619 (0.614–0.624)	0.333 (0.329–0.338)	0.434 (0.429–0.440)
	XGBoost	0.686 (0.681–0.693)^a^	0.411 (0.401–0.421)^a^	0.673 (0.664–0.682)	0.590 (0.585–0.595)	0.334 (0.330–0.338)	0.446 (0.441–0.452)
	TabNet	0.670 (0.664–0.677)	0.393 (0.383–0.403)	0.674 (0.664–0.683)	0.565 (0.559–0.570)	0.321 (0.317–0.325)	0.435 (0.429–0.440)
	TabNet^self^	0.676 (0.670–0.682)^b^	0.396 (0.386–0.405)^b^	0.626 (0.615–0.636)	0.628 (0.623–0.634)	0.339 (0.334–0.344)	0.440 (0.434–0.446)
SXT	LR	0.666 (0.660–0.673)	0.467 (0.458–0.478)	0.568 (0.559–0.577)	0.674 (0.669–0.680)	0.391 (0.386–0.397)	0.463 (0.457–0.470)
	XGBoost	0.701 (0.695–0.707)^a^	0.524 (0.514–0.534)^a^	0.660 (0.651–0.669)	0.618 (0.612–0.623)	0.389 (0.384–0.393)	0.489 (0.483–0.495)
	TabNet	0.685 (0.678–0.691)	0.497 (0.487–0.508)	0.629 (0.620–0.639)	0.635 (0.630–0.641)	0.389 (0.384–0.394)	0.480 (0.475–0.487)
	TabNet^self^	0.693 (0.687–0.699)^b^	0.503 (0.492–0.513)^b^	0.637 (0.628–0.646)	0.641 (0.635–0.646)	0.395 (0.388–0.402)	0.488 (0.480–0.496)
CIP	LR	0.789 (0.784–0.794)	0.590 (0.580–0.599)	0.601 (0.592–0.611)	0.832 (0.827–0.836)	0.539 (0.532–0.546)	0.569 (0.561–0.575)
	XGBoost	0.811 (0.806–0.816)^a^	0.617 (0.608–0.627)^a^	0.727 (0.718–0.736)	0.749 (0.744–0.753)	0.487 (0.481–0.492)	0.583 (0.577–0.589)
	TabNet	0.798 (0.793–0.802)	0.576 (0.566–0.586)	0.729 (0.721–0.738	0.730 (0.725–0.734)	0.469 (0.464–0.475)	0.571 (0.566–0.577)
	TabNet^self^	0.800 (0.796–0.805)^b^	0.584 (0.575–0.595)^b^	0.726 (0.718–0.734)	0.737 (0.733–0.742)	0.504 (0.498–0.512)	0.595 (0.588–0.603)
LVX	LR	0.791 (0.786–0.796)	0.592 (0.582–0.602)	0.632 (0.623–0.641)	0.809 (0.805–0.813)	0.524 (0.518–0.530)	0.573 (0.566–0.579)
	XGBoost	0.814 (0.810–0.819)^a^	0.624 (0.614–0.634)^a^	0.710 (0.702–0.719)	0.769 (0.765–0.773)	0.506 (0.500–0.511)	0.591 (0.585–0.597)
	TabNet	0.803 (0.798–0.808)	0.597 (0.587–0.608)	0.725 (0.718–0.734)	0.737 (0.732–0.741)	0.478 (0.473–0.483)	0.576 (0.571–0.582)
	TabNet^self^	0.808 (0.803–0.813)^b^	0.606 (0.597–0.617)^b^	0.713 (0.705–0.721)	0.764 (0.760–0.769)	0.527 (0.520–0.535)	0.606 (0.598–0.614)

To evaluate the generalizability of our models, we additionally performed validation on an independent cohort of patients with uncomplicated UTI specimens. We used the trained XGBoost and TabNet^self^ models, as these achieved the best and second-best scores during temporal validation on the complicated UTI specimens. We present results for all specimens (*n* = 15,608), as well as results for a smaller subset (*n* = 3941) which is equivalent to the test set evaluated in Kanjilal et al.^5^, allowing for direct comparison. For all antibiotics, AUROC and AUPRC are lower for the uncomplicated cohort than the complicated cohort; however, they are comparable to those reported in the previous study, despite the previous study being specifically trained on uncomplicated UTI, and this study being trained on potentially complicated UTI (Table 3).

**Table 3:** Performance metrics, alongside 95% confidence intervals, for antibiotic resistance prediction for patients with uncomplicated UTI (bolded values denote best scores for AUROC and AUPRC comparing XGBoost and TabNet^self^ models).

		TabNet^self^		XGBoost		Kanjilal et al.^5^
		AUROC	AUPRC	AUROC	AUPRC	AUROC
NIT	All	0.575 (0.563–0.587)	0.172 (0.159–0.185)	0.593 (0.580–0.605)	0.186 (0.173–0.200)	
	Test	0.543 (0.517–0.566)	0.145 (0.128–0.169)	0.559 (0.534–0.584)	0.162 (0.142–0.187)	0.56 (0.53–0.59)
SXT	All	0.603 (0.594–0.613)	0.301 (0.289–0.315)	0.612 (0.603–0.621)	0.318 (0.305–0.331)	
	Test	0.591 (0.571–0.610)	0.292 (0.268–0.320)	0.589 (0.570–0.608)	0.294 (0.268–0.322)	0.59 (0.57–0.62)
CIP	All	0.670 (0.651–0.688)	0.249 (0.225–0.276)	0.676 (0.659–0.694)	0.254 (0.230–0.281)	
	Test	0.646 (0.611–0.679)	0.244 (0.199–0.294)	0.639 (0.606–0.673)	0.245 (0.202–0.294)	0.64 (0.60–0.68)
LVX	All	0.662 (0.644–0.678)	0.228 (0.204–0.255)	0.667 (0.649–0.685)	0.244 (0.220–0.273)	
	Test	0.639 (0.604–0.671)	0.256 (0.215–0.304)	0.623 (0.586–0.657)	0.266 (0.221–0.314)	0.64 (0.60–0.68)

Due to the ambiguity in how ethnicity/race was documented, we proceeded to conduct an extra experiment. Specifically, we ran the best performing model, XGBoost, excluding the ethnicity/race feature. The outcomes obtained on the test sets fell within the 95% confidence intervals (CIs) of the original models (which encompassed ethnicity/race as a feature). The corresponding *p*-values were 0.468, 0.148, 0.023, and <0.001 for NIT, SXT, CIP, and LVX, respectively, across 1000 bootstrapped iterations. Full numerical results can be found in Supplementary Table 9.

Overall, the results show promise that model-assigned probabilities of antibiotic resistance can differentiate potentially complicated UTI specimens resistant to one antibiotic and susceptible to another at the single-patient level. Additionally, we found that the trained models can be generalized to uncomplicated UTI specimens, thus motivating further development of algorithmic decision-support for antibiotic recommendations.

### Feature importance

Beyond solely classifying samples, all models can provide information on which features were most important for determining resistance (in the form of coefficients for logistic regression, and importance scores for TabNet and XGBoost models). For all models, prior antibiotic resistance and prior antibiotic exposure, across different time frames, were generally found to be the most important features in predicting resistance to each antibiotic. This included previous use of common antibiotics (both the outcome antibiotics considered in our study, as well as other antibiotics) for UTI treatment such as fluoroquolines (e.g. CIP and LVX), cephalosporins (e.g. cefepime, ceftriaxone, cefpodoxime), and penicillins (e.g. amoxicillin). Similarly, previous UTI history (i.e. if any – susceptible or non-susceptible – isolates of infecting pathogens, such as *E.coli*, were found within previous patient specimens), was found to be predictive of resistance. For the second-line antibiotics (such as CIP and LVX), resistance to one was predictive of resistance to the other, which is expected, as both antibiotics belong to the same family of antibacterial agents. Additionally, comorbidities, including those categorized as paralysis and renal, were ranked highly across all antibiotics and models. Previous stays in a long-term care facility (skilled nursing facility) and whether a patient had undergone a surgical procedure were also considered as highly predictive factors. A full summary of the top 30 features used in prediction for each model, alongside their importance scores, can be found Supplementary Tables 5, 6, 7, 8.

**Fig. 1:**
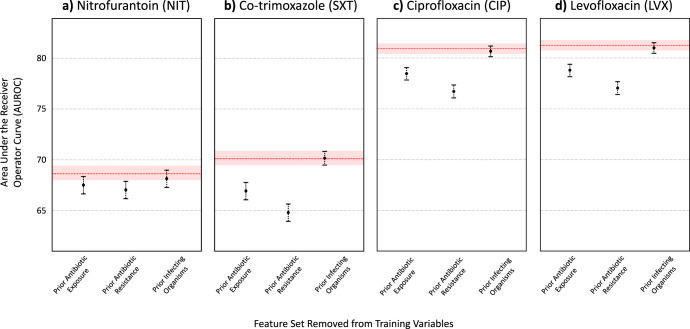
AUROC of XGBoost models trained without the feature set labeled on the *x*-axis, with error bars representing 95% CIs. The red line depicts the AUROC for the model trained on all features, with the red shaded region representing 95% CIs. Results shown for (**a**) Nitrofurantoin (NIT), (**b**) Co-trimoxazole (SXT), (**c**) Ciprofloxacin (CIP), and (**d**) Levofloxacin (LVX).

**Fig. 2:**
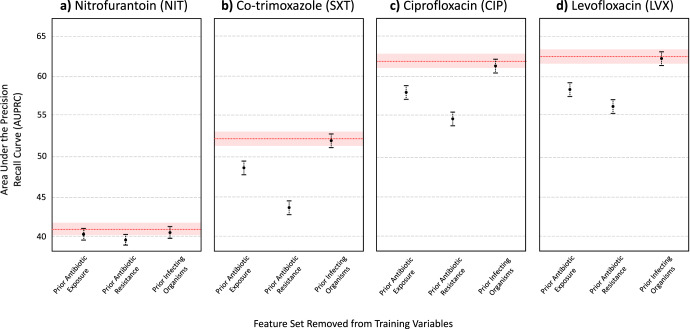
AUPRC of XGBoost models trained without the feature set labeled on the *x*-axis, with error bars representing 95% CIs. The red line depicts the AUPRC for the model trained on all features, with the red shaded region representing 95% CIs. Results shown for (**a**) Nitrofurantoin (NIT), (**b**) Co-trimoxazole (SXT), (**c**) Ciprofloxacin (CIP), and (**d**) Levofloxacin (LVX).

Finally, we grouped features into sets that corresponded to general risk factor domains that were found to be associated with resistance. Using the XGBoost model architecture, we evaluated the decrease in predictive performance when a particular feature set was left out of training (Figs. 1 and 2 for AUROC and AUPRC scores, respectively). In general, prior antibiotic resistance was found to be the most important feature set in predicting antibiotic resistance. When left out, AUPRC decreased by 0.0199 (0.0142- 0.0265), 0.0877 (0.0800–0.0947), 0.0695 (0.0623–0.0759), and 0.0631 (0.0566–0.0692), for NIT, SXT, CIP, and LVX, respectively (for all antibiotics, decrease in AUPRC was found to be significant when compared to XGBoost models trained with all feature sets; *p* < 0.001, determined using 1000 bootstrap samples). Prior antibiotic exposure was also found to be an important feature set, as AUPRC decreased by 0.0089 (0.0035–0.0142), 0.0401 (0.0035–0.0142), 0.0383 (0.0327–0.0441), and 0.0414 (0.0352–0.0472), for NIT, SXT, CIP, and LVX, respectively (*p* = 0.001 for NIT, and *p* < 0.001 for SXT, CIP, and LVX models). This aligns with the feature rankings obtained through the importance scores/coefficients quantified by each trained model. Although the absence of prior infecting organism features (i.e. prior UTI history) in training decreased predictive performance (AUPRC scores decreased by 0.0049 [0.0019–0.0072], 0.0015 [−0.0019–0.0052], 0.0038 [0.0000–0.0079], and 0.0034 [−0.0003–0.0068] for NIT, SXT, CIP, and LVX, respectively), changes in AUPRC scores were not generally found to be statistically significant (*p* = 0.001, 0.403, 0.059, 0.062 for NIT, SXT, CIP, and LVX models, respectively). Similar patterns were found for AUROC scores. Full numerical results can be found in Supplementary Tables 10, 11, and 12.

## Discussion

Our analysis of EHRs from a substantial patient cohort has showcased the predictive potential of interpretable ML methods in identifying antibiotic resistance within potentially complicated UTIs. It is crucial to emphasize that, while our findings offer valuable insights, our proof of concept underscores the need for further validation studies before machine learning algorithms can be widely embraced for reducing treatment mismatches and enabling personalized treatment recommendations.

We found that both XGBoost and TabNet models surpassed logistic regression, implying the presence of non-linear trends and interactions that cannot be adequately captured through linear combinations of input features. The best performances were achieved using XGBoost, however, TabNet, when combined with self-supervised learning (TabNet^self^), also achieved comparably high performance. The potential superiority of XGBoost over TabNet may be a result of its ensemble architecture, whereby the predictions of multiple models are combined^15^, improving generalization error. Additionally, decision tree-based techniques, like XGBoost, have historically exhibited superior performance (over neural network-based architectures) when dealing with tabular data containing a mix of continuous and categorical attributes^16,17^. However, there are advantages to using a neural network-based architecture (such as TabNet) including (1) it can be used in combination with transfer learning and self-supervised learning, whereas tree-based algorithms typically depend on the availability of the entire dataset, making transfer learning infeasible; and (2) it can be used for image recognition tasks, as well as natural language problems, which XGBoost is typically not appropriate for.

The ability of a TabNet model to use transfer learning may be of particular importance in a clinical context, as it enables predictions to be updated over time. As our findings are specific to a specific patient cohort (data from MGH and BWH during 2007–2016), results may differ for other patient cohorts and hospital locations due to variations in the prevalence of antimicrobial resistance, clinical practice, and patient characteristics. Furthermore, as antimicrobial resistance is an evolving phenomenon, new resistance mechanisms can emerge over time, rendering existing models outdated or less accurate. Through the acquisition of more/new data, the weights of a neural network-based model can be finetuned, rather than fully retrained, in order to keep models up-to-date^10^.

We found that we achieved better overall performance on the complicated UTI cohort than the uncomplicated UTI cohort. This may be because of greater hospital exposure (and related factors) in the complicated UTI cohort, making it easier to predict antibiotic susceptibility, compared to the uncomplicated cohort. Although we trained our models on the complicated UTI cohort, we still achieved comparable AUROC scores (when validating on the uncomplicated cohort), as a previous study^5^ which trained and tested models using exclusively data from an uncomplicated UTI cohort. This may be due to the greater amount of data available for training, as ML models (and particularly, neural networks) typically need a large amount of training data to achieve generalizability.

Since the training data was imbalanced, we used threshold adjustment to determine the final susceptibility label and optimize the balance between sensitivity and specificity. However, a model’s output can be biased on its training dataset, which subsequently affects the derived optimal threshold^10^. Consequently, thresholds appropriate for one dataset might not be applicable to another with differing distributions. Hence, exploring the ideal decision threshold is crucial, as consistent sensitivity/specificity scores across testing groups is necessary for model reliability^10^. To ensure real-world effectiveness, future experiments could gradually adjust thresholds during deployment to align with real-time distributions, for standardized predictive performance. Alternatively, developers might balance data during preprocessing to mitigate imbalance issues and circumvent threshold adjustment. We didn’t choose this latter approach, as we wanted to retain true prevalence rates during model development.

On a similar note, we also appreciate that the probability of antibiotic susceptibility is a useful measurement, as opposed to thresholding to a binary label. We used a binary classification to align with the CLSI clinical breakpoints used in the AMR-UTI dataset; however, probability can also be used as a final output for tasks where appropriate. For instance, in scenarios where changes in susceptibility within a population can arise, the use of minimum inhibitory concentration (MIC) can be suitable^18^. MIC has previously demonstrated success in various resistance prediction tasks using methodologies like logistic regression, Random Forest, and XGBoost^18–21^, making it a suitable outcome to explore in relevant contexts.

In terms of feature importance, prior antibiotic resistance and antibiotic exposure was found to be highly predictive of resistance across all antibiotics. This is expected as antibiotic resistance has been found to be associated with previous UTI occurrences and their resistances^3,22,23^. These features were ranked highly across multiple time frames preceding specimen collection, suggesting both short- and long-term associations with resistance. In our investigations, times were binned; however, future studies may benefit from keeping a higher degree of granularity, as well as using models more suitable for time-series analysis/forecasting (e.g. convolutional neural network, long short-term memory network), to better capture temporal associations. Other antibiotic exposures (other than the antibiotic being tested) were also ranked highly amongst the models. This is consistent with previous studies^24–27^, where a specific antibiotic exposure was found to both directly select for strains resistant to it, as well as indirectly select for resistance to other antibiotics (e.g. through common co-occurrence). For example, previous studies have found that low ciprofloxacin levels open up the mutant selection window, leading to rapid selection of resistant subpopulations^27^. Additionally, metabolic mutations have been observed to arise in response to antibiotic treatment, including ciprofloxacin^28^. These mutations subsequently confer resistance and are widespread among clinical pathogens. Notably, in our assessments, the history of ciprofloxacin use or previous resistance to ciprofloxacin emerged as important features across all models. This highlights the critical importance of administering antibiotics at proper dosages and underscores the possible consequences of administering insufficient dosages, which might facilitate the survival and propagation of antibiotic-resistant mutants. While this falls outside of our current investigation, it presents an intriguing prospect for follow-up studies to delve into using machine learning.

In addition to antibiotic-related features, comorbidities, including those categorized as paralysis and renal, were also commonly ranked as being important for determining resistance. These have previously been found to be associated with UTIs – patients with prior kidney diseases are at higher risk of developing UTIs;^29^ and patients with paralysis may have had a catheter-associated UTI (CAUTI), as catheters have been found to be a common cause of healthcare-associated UTIs^22,26,30^. Both of these factors can lead to recurrent UTIs; and thus, lead to antibiotic resistance due to prior exposure/use. This may also reflect why stays in a long-term care facility or undergoing a surgical procedure were also ranked as highly predictive, as patients may require the use of a catheter^26,31^. Additionally, patients undergoing long surgical procedures may have postoperative urinary retention, which can also lead to a UTI^32^.

Given the extensive scope of patients and clinical factors associated with CAUTIs and healthcare-associated UTIs, forthcoming research could concentrate specifically on investigating CAUTIs or distinguishing between community-acquired UTIs and hospital-acquired UTIs. Similarly, the training dataset may include individuals with conditions such as asymptomatic bacteriuria (ASB), which could potentially hinder model performance. To enhance the precision of future models, it’s advisable to either exclude ASB patients from the training data, integrate an extra marker addressing this aspect during training, or create a separate model focused solely on ASB patients. These more refined cohorts can address specialized tasks and provide more targeted insights.

With respect to ethnicity/race, the AMR-UTI dataset classified each patient as either “white” or “non-white”. However, the use of a binary label for ethnicity/race can pose challenges, as models can inherit biases from the data they are trained on^33,34^. It’s critically important that the features used in the models neither introduce bias in favor of or against individuals or groups based on the terminology or categories employed^35,36^. Additionally, ethnicity can play a significant role in predicting specific diagnoses, prognoses, and treatment recommendations. Therefore, achieving higher prediction accuracy in clinical tasks might necessitate a more nuanced approach to capture the diverse facets of ethnicity^34^. When we conducted a comparison between models trained with and without the inclusion of ethnicity/race as a feature, we observed that the outcomes achieved on the test sets were consistent with each other and fell within their respective 95% confidence intervals (CIs). Consequently, in our specific scenario, where ethnicity/race was presented in a binary manner and did not significantly contribute to the predictive task, it appears unnecessary to incorporate it into the final models. Moreover, as machine learning gains prominence within clinical realms, there is a greater need for meticulous consideration of how ethnicity/race is captured in data and integrated into machine learning algorithms. This attention is essential to prevent inadvertent reinforcement of existing biases and to achieve a nuanced representation that can facilitate enhanced prediction accuracy.

We also recognize that the AMR-UTI dataset offers a constrained view of the comprehensive information found in electronic health record (EHR) systems. Notably, substantial segments of EHR data, such as patient symptoms (like dysuria, urinary frequency, costovertebral tenderness), treatment-related details (including antimicrobial dosage, duration, Intravenous [IV] vs oral administration), and lifestyle/environmental factors (like travel history, diet, physical activity), are not fully encompassed by the AMR-UTI dataset. Furthermore, details like antibiotic purchase history and the status of antimicrobial dispensation (ordered vs consumed) were absent. However, these details are crucial for precise treatment evaluation, informed clinical decisions, antimicrobial stewardship, and overall patient safety. A previous study utilizing ML to predict antibiotic susceptibility highlighted the significant predictive power of antibiotic purchase history^3^. Also, because the AMR-UTI dataset lacked empirical clinician prescriptions for patients with potentially complex UTIs, our algorithm wasn’t designed to propose specific treatments (this presents a logical progression for future studies). Hence, forthcoming investigations should consider integrating other crucial features into these models while collaborating closely with domain experts and clinicians.

Future studies can also consider training one multilabel classifier/learning combinations of resistances, rather than training multiple binary classifiers. This may be beneficial, as resistances to one antibiotic can affect the resistance to others; and thus, a single model that considers all antibiotics can account for the fact that patients can have multiple resistances. Additionally, for tasks where very large models need to be used, training multiple binary models can overwhelm computing power. However, it should be noted that multilabel tasks often require more data to confidently differentiate between all classes, especially for challenging clinical tasks.

Finally, prompt initiation of appropriate antimicrobial treatment is crucial for effective infection management. However, in stable patient cases, the option of delaying treatment while awaiting susceptibility results arises^37^. These results typically require an extra 24 h, which raises questions about the relevance of ML-based algorithms in such scenarios. While these algorithms provide fast predictions, their usefulness might be challenged in these specific situations, as the delay from waiting could make their predictions seem redundant. In such cases, the traditional approach of waiting for results might suffice if the patient’s condition is stable. However, there are instances where rapid predictions from ML models remain valuable, particularly in cases of patient instability, clinical urgency, or potential rapid disease progression (which can be the case for potentially complicated UTI infections). These models can offer quick insights and could be used in tandem with clinical judgment for interim decisions while awaiting susceptibility results. Ultimately, the decision to use ML algorithms should account for the clinical context, patient condition, and urgency. Despite potential treatment delays due to testing, these algorithms could still prove beneficial, especially when swift decisions are essential.

## Methods

### Dataset, features, and preprocessing

We trained and tested our models using the AMR-UTI dataset^38,39^, which is a freely accessible dataset of over 80,000 patients with UTIs presenting between 2007 and 2016 at Massachusetts General Hospital (MGH) and Brigham & Women’s Hospital (BWH) (approved by the Institutional Review Board of Massachusetts General Hospital with a waived requirement for informed consent). Our analysis centered on individuals with potentially complicated UTIs, encompassing a total of 101,096 samples. This group represented a broader cohort that did not fulfill the criteria outlined in the study by Kanjilal et al.^5^, which focused on uncomplicated UTIs. Our cohort included numerous patients with complex infections that might necessitate treatment involving a variety of antibiotics. We included all specimens that were tested for any one or combination of the local first-line agents – nitrofurantoin (NIT) or co-trimoxazole (SXT) – or second-line agents – ciprofloxacin (CIP) or levofloxacin (LVX).

To allow for direct comparison with findings in Kanjilal et al.^5^, we used a similar feature set and data filtering protocol as those used for the uncomplicated UTI cohort. Thus, each observation includes corresponding urine specimens which were sent to the clinical microbiology laboratory for assessment of AMR. Full de-identified feature sets include (1) the antimicrobial susceptibility profile, (2) previous specimen features useful for AMR prediction, and (3) basic patient characteristics.

With respect to the antimicrobial susceptibility profile, the raw data received from the clinical microbiology laboratory included the identity of the infecting pathogen, alongside the results of susceptibility testing to various antibiotics. These were determined by minimum inhibitory concentration (MIC) and disk diffusion (DD) based methods, and the numerical results of these tests were transformed into categorical phenotypes using the published 2017 Clinical and Laboratory Standards Institute (CLSI) clinical breakpoints. This conversion resulted in three phenotypes: susceptible (S), intermediate (I), and resistant (R). The AMR-UTI dataset treated both intermediate and resistant phenotypes as resistant, which is typically in-line with what is done in clinical practice^38^. We adopt the same simplifying approach.

EHR data included patient demographic features such as age and ethnicity, prior antibiotic resistance, prior antibiotic exposures, prior infecting organisms, comorbidity diagnoses, where the specimen was collected (inpatient, outpatient, emergency room [ER], intensive care unit [ICU]), colonization pressure (rate of resistance to that agent within a specified location and time period), prior visits to skilled nursing facilities, infections at other sites (other than urinary), and prior procedures. Colonization pressure was computed as the proportion of all urinary specimens resistant to an antibiotic in the period ranging from 7 to 90 days before the date of specimen collection (for a given specimen), across 25 antibiotics. Resistance rates were recorded for three location hierarchies – specimens collected at the same floor/ward/clinic, specimens collected at the same hospital (MGH or BWH) and department type (inpatient, outpatient, ICU, ER), and all specimens collectively. Infections at other sites were included for those patients who had other specimens collected (on the same day as the urinary specimen) from other infection sites. Antibiotic exposures, prior resistance, prior organism, laboratory data, comorbidities, and prior hospitalizations were recorded for 14, 30, 90, and 180 days preceding specimen collection. These data do not include information on the dose or duration of antibiotic therapy, urinalysis results, drug allergies, or data for patient encounters outside of MGH and BWH. Empiric clinician prescriptions for patients diagnosed with complicated UTIs were not available in the dataset.

All categorical variables were one-hot encoded, totalling 787 features used for model development. A full list of features used can be found in Supplementary Table 1. Missing values were already addressed within the dataset (as most features are binary, 1 indicates the presence of an observed element and 0 indicates that an element was not observed, including those cases where data is missing). Detailed documentation on data inclusion, exclusion, features, feature descriptions, and analytic protocols used for the AMR-UTI dataset can be found in the PhysioNet repository (https://physionet.org/content/antimicrobial-resistance-uti/1.0.0/).

To train and test our models, we used temporal evaluation, where models were trained on data from patients who submitted urine specimens between 2007 and 2013; and then tested on specimens submitted between 2014 and 2016. By temporally separating the data between training and test sets, we can emulate the real-world implementation of such a forecasting method for AMR. From the initial training data, we used 90% for model development, hyperparameter selection, and model training, and the remaining 10% for continuous validation and threshold adjustment of results. After successful model development and training, the held-out test set was used to evaluate the performance of the final models. Using the same features and preprocessing protocol, we additionally evaluated our method on the held-out uncomplicated UTI patient cohort (15,806 specimens). This is the same dataset used in Kanjilal et al.^5^, allowing us to evaluate the generalizability of our models, as well as directly compare results to those from a previous benchmark.

Regarding ethnicity/race, the AMR-UTI dataset adopted a binary approach, classifying each patient as either “white” or “non-white”. In instances where race isn’t recorded, which accounts for 3% of cases, the feature defaults to “non-white”. However, the use of a binary label for ethnicity/race can pose challenges, as it may not be all-encompassing and could inadvertently perpetuate existing biases^33–36^. This is further elaborated on in the Discussion. To ensure direct comparability with Kanjilal’s study^5^, we will train models that include the ethnicity/race feature. Nevertheless, we will also explore models that exclude this feature in our evaluation.

It is also essential to emphasize that the AMR-UTI dataset lacks information to ascertain whether patients had other conditions, such as asymptomatic bacteriuria (ASB), which often leads to positive urine cultures. However, ASB is typically not an appropriate indication for antibiotic therapy^13,40^. Consequently, there is a possibility that these patients might inadvertently be included in the training and validation cohorts without explicit identification.

### Machine learning architecture

We trained logistic regression, XGBoost, and TabNet models to predict the probability that a specimen would be resistant/non-susceptible to NIT, SXT, CIP, or LVX. All models can handle tabular data consisting of both continuous and categorical features, and additionally, enable interpretability by quantifying the contributions of each feature to the trained model.

Logistic Regression (LR) is widely accepted in clinical decision-making, and additionally, has previously been shown to perform the best when evaluating uncomplicated UTI specimens, which were obtained using the same protocol as the complicated UTI cohort used in our study^5^. This makes it an appropriate benchmark for comparison to more complex models.

XGBoost is an optimized distributed gradient boosting library, based on decision trees (DTs), which has been found to achieve state-of-the-art results on many machine learning problems, especially those using structured or tabular datasets (as we use in our study). DT-based algorithms have also been shown to be effective at predicting AMR from clinical data^3^.

TabNet is a machine learning model designed for tabular data, which utilizes “sequential attention” to improve model performance and interpretability. We train it using both a traditional supervised learning approach, as well as a pre-trained approach. Specifically, we present results for a separate set of TabNet models which have been pre-trained using self-supervised learning (via unsupervised representation learning). Here, we train a decoder network to reconstruct the original tabular features from the encoded representations, through the task of predicting missing feature columns from the others. This ultimately results in an improved encoder model to be used during the main supervised learning task. Details about the TabNet architecture and the self-supervised method used can be found in the original TabNet publication^8^.

Details on model implementations and software packages used can be found in the Supplementary Methods section of the Supplementary Material.

### Evaluation metrics

For the evaluation of the trained models, performance metrics including sensitivity, specificity, area under the receiver operator characteristic curve (AUROC), area under the precision-recall curve (AUPRC), positive predictive value (PPV), and F1-score are presented. These metrics are accompanied by their respective 95% confidence intervals (CIs), which are calculated from 1000 bootstrapped samples drawn from the test set. The reported scores fall within the range of [0, 1], where values closer to 1 indicate better performance. Tests of significance (*p*-values) comparing model performances are calculated by evaluating how many times one model performs better than other models across 1000 pairs of bootstrapped iterations.

### Hyperparameter optimization and threshold adjustment

For each model developed, hyperparameter values were determined through standard five-fold cross-validation and grid search using respective training sets. This ensured that different combinations of hyperparameter values were evaluated on as much data as possible to provide the best estimate of model performance on unseen data. This allowed us to choose the optimal settings for model training. We chose the hyperparameter set based on the best AUPRC scores to account for the relative imbalance in the dataset. Details on the hyperparameter values used in the final models can be found in Supplementary Table 3.

As the raw output of each classifier is a probability of class membership, a threshold is needed to map each specimen to a particular class label. For binary classification, the default threshold is typically 0.5 (values equal to or greater than 0.5 are mapped to one class and all other values are mapped to the other); however, this threshold can lead to poor performance, especially when the dataset used to train a model has a large class imbalance^10^. This is seen in our training sets, as there are far fewer non-susceptible cases than susceptible ones, across all antibiotics. Thus, we used a grid search to adjust the decision boundary used for identifying non-susceptible specimens, to improve detection rates at the time of testing. We chose to optimize for balanced sensitivity and specificity to ensure that we can identify resistant samples (to avoid unsuccessful treatment), as well as ensure that samples which are susceptible get treated with the appropriate local first-line antibiotic (and avoid having to potentially use more antibiotics), respectively. The optimal thresholds were determined through a grid search using the validation dataset, and were then applied to the results obtained on the held-out test set. Final threshold values used can be found in Supplementary Table 4.

### Reporting summary

Further information on research design is available in the Nature Research Reporting Summary linked to this article.

### Supplementary information

Supplementary Material

Reporting Summary

## Data Availability

The AMR-UTI data can be downloaded from: https://physionet.org/content/antimicrobial-resistance-uti/1.0.0/.

## Code availability

The code used to run all models is provided in the Supplementary Material.
