# Supplementary material for: Interpretable machine learning-based decision support for prediction of antibiotic resistance for complicated urinary tract infections
Source: NPJ Antimicrob Resist. 2023 Nov 2;1:14. doi: 10.1038/s44259-023-00015-2 (PMC11057209; doi:10.1038/s44259-023-00015-2)
Supplement: Supplementary file 2 — Reporting Summary [file 44259_2023_15_MOESM2_ESM.pdf]

nature portfolio

Corresponding author(s): Jenny Yang
Last updated by author(s): Aug 10, 2023

Reporting Summary

Nature Portfolio wishes to improve the reproducibility of the work that we publish. This form provides structure for consistency and transparency in reporting. For further information on Nature Portfolio policies, see our Editorial Policies and the Editorial Policy Checklist.

Statistics

For all statistical analyses, confirm that the following items are present in the figure legend, table legend, main text, or Methods section.

n/a	Confirmed	
☐	☒	The exact sample size (*n*) for each experimental group/condition, given as a discrete number and unit of measurement
☐	☒	A statement on whether measurements were taken from distinct samples or whether the same sample was measured repeatedly
☐	☒	The statistical test(s) used AND whether they are one- or two-sided<br>*Only common tests should be described solely by name; describe more complex techniques in the Methods section.*
☒	☐	A description of all covariates tested
☐	☒	A description of any assumptions or corrections, such as tests of normality and adjustment for multiple comparisons
☐	☒	A full description of the statistical parameters including central tendency (e.g. means) or other basic estimates (e.g. regression coefficient) AND variation (e.g. standard deviation) or associated estimates of uncertainty (e.g. confidence intervals)
☐	☒	For null hypothesis testing, the test statistic (e.g. *F*, *t*, *r*) with confidence intervals, effect sizes, degrees of freedom and *P* value noted<br>*Give P values as exact values whenever suitable.*
☒	☐	For Bayesian analysis, information on the choice of priors and Markov chain Monte Carlo settings
☐	☒	For hierarchical and complex designs, identification of the appropriate level for tests and full reporting of outcomes
☒	☐	Estimates of effect sizes (e.g. Cohen's *d*, Pearson's *r*), indicating how they were calculated

Our web collection on statistics for biologists contains articles on many of the points above.

Software and code

Policy information about availability of computer code

Data collection	No software was used to collect data. Data can be downloaded online at: https://physionet.org/content/antimicrobial-resistance-uti/1.0.0/
Data analysis	Models were implemented using Python (v3.6.9). XGBoost baseline models were implemented using the XGBoost library (v1.3.3). Logistic regression models were implemented using sklearn (v0.24.1). TabNet models were implemented using the pytorch-tabnet package (4.0) and torch (v1.7.0) All models were run using an Intel Xeon E-2146G Processor (CPU: 6 cores, 4.50 GHz max frequency).

For manuscripts utilizing custom algorithms or software that are central to the research but not yet described in published literature, software must be made available to editors and reviewers. We strongly encourage code deposition in a community repository (e.g. GitHub). See the Nature Portfolio guidelines for submitting code & software for further information.

Data

Policy information about availability of data

All manuscripts must include a data availability statement. This statement should provide the following information, where applicable:

- Accession codes, unique identifiers, or web links for publicly available datasets
- A description of any restrictions on data availability
- For clinical datasets or third party data, please ensure that the statement adheres to our policy

Data can be downloaded online at: https://physionet.org/content/antimicrobial-resistance-uti/1.0.0/

Research involving human participants, their data, or biological material

Policy information about studies with human participants or human data. See also policy information about sex, gender (identity/presentation), and sexual orientation and race, ethnicity and racism.

Reporting on sex and gender	This data was unavailable in the repository. We have stated this in the manuscript.
Reporting on race, ethnicity, or other socially relevant groupings	Regarding ethnicity/race, the AMR-UTI dataset adopted a binary approach, classifying each patient as either "white" or "non-white". In instances where race isn't recorded, which accounts for 3% of cases, the feature defaults to "non-white". We refer to this demographic feature as ethnicity/race because in the original manuscript (which released this dataset), this feature was referred to as ethnicity/race. We have discussed the limitations and considerations involved with using this feature within machine learning.
Population characteristics	Patients in the training set cohort had a median age of 64 years (IQR 44-76), with 72.9% of patients self-identifying as white; the validation cohort also had a median age of 64 (44-76), with 73.6% self-identifying as white; and the test cohort had a median age of 64 (45-76), with 72.7% self-identifying as white. It should be noted that demographic information on the sex of patients in the complicated UTI cohort was not available.
Recruitment	The AMR-UTI dataset on PhysioNet is a freely accessible dataset of over 80,000 patients with UTIs presenting between 2007 and 2016 at Massachusetts General Hospital (MGH) and Brigham & Women's Hospital (BWH).
Ethics oversight	This study was approved by the Institutional Review Board (IRB) of Massachusetts General Hospital with a waived requirement for informed consent.

Note that full information on the approval of the study protocol must also be provided in the manuscript.

Field-specific reporting

Please select the one below that is the best fit for your research. If you are not sure, read the appropriate sections before making your selection.

☒ Life sciences ☐ Behavioural & social sciences ☐ Ecological, evolutionary & environmental sciences

For a reference copy of the document with all sections, see nature.com/documents/nr-reporting-summary-flat.pdf

Life sciences study design

All studies must disclose on these points even when the disclosure is negative.

Sample size	Using the AMR-UTI dataset, we included all individuals with potentially complicated UTIs, encompassing a total of 101,096 samples.
Data exclusions	No data was excluded. All data was used for either model training, validation, or testing.
Replication	We validated results temporally, and on a separately defined cohort. We bootstrapped our method (using 1000 iterations) to obtain appropriate confidence intervals for all outcomes.
Randomization	Allocation of patient specimens for training and validation were based on time of hospital presentation/stay (to allow for temporal validation) and whether UTIs were considered complicated or uncomplicated (to allow for generalizability testing across the different groups).
Blinding	Blinding is not applicable, as this study compared machine learning results across all patients.

Reporting for specific materials, systems and methods

We require information from authors about some types of materials, experimental systems and methods used in many studies. Here, indicate whether each material, system or method listed is relevant to your study. If you are not sure if a list item applies to your research, read the appropriate section before selecting a response.

Materials & experimental systems

n/a	Involved in the study	
☒	☐	Antibodies
☒	☐	Eukaryotic cell lines
☒	☐	Palaeontology and archaeology
☒	☐	Animals and other organisms
☐	☒	Clinical data
☒	☐	Dual use research of concern
☒	☐	Plants

Methods

n/a	Involved in the study	
☒	☐	ChIP-seq
☒	☐	Flow cytometry
☒	☐	MRI-based neuroimaging

Clinical data

Policy information about clinical studies

All manuscripts should comply with the ICMJE guidelines for publication of clinical research and a completed CONSORT checklist must be included with all submissions.

Clinical trial registration	NA
Study protocol	The data repository can be found: https://physionet.org/content/antimicrobial-resistance-uti/1.0.0/
Data collection	The AMR-UTI dataset is a freely accessible dataset of over 80,000 patients with UTIs presenting between 2007 and 2016 at Massachusetts General Hospital (MGH) and Brigham & Women's Hospital (BWH).
Outcomes	Based on the susceptibility testing performed, we included outcomes for the four antibiotics for which data was available.
